# Beneficial soil bacterium *Bacillus subtilis* (GB03) augments salt tolerance of white clover

**DOI:** 10.3389/fpls.2014.00525

**Published:** 2014-10-08

**Authors:** Qing-Qing Han, Xin-Pei Lü, Jiang-Ping Bai, Yan Qiao, Paul W. Paré, Suo-Min Wang, Jin-Lin Zhang, Yong-Na Wu, Xiao-Pan Pang, Wen-Bo Xu, Zhi-Liang Wang

**Affiliations:** ^1^State Key Laboratory of Grassland Agro-ecosystems, College of Pastoral Agriculture Science and Technology, Lanzhou UniversityLanzhou, China; ^2^Gansu Key Laboratory of Crop Genetic and Germplasm Enhancement, Gansu Provincial Key Laboratory of Arid Land Crop Science, College of Agronomy, Gansu Agricultural UniversityLanzhou, China; ^3^Department of Chemistry and Biochemistry, Texas Tech UniversityLubbock, TX, USA

**Keywords:** *Bacillus subtilis*, white clover, salt tolerance, MDA, osmotic potential, Na^+^ accumulation

## Abstract

Soil salinity is an increasingly serious problem worldwide that reduces agricultural output potential. Selected beneficial soil bacteria can promote plant growth and augment tolerance to biotic and abiotic stresses. *Bacillus subtilis* strain GB03 has been shown to confer growth promotion and abiotic stress tolerance in the model plant *Arabidopsis thaliana*. Here we examined the effect of this beneficial soil bacterium on salt tolerance in the legume forage crop, white clover. Plants of white clover (*Trifolium repens* L. cultivar Huia) were grown from seeds with or without soil inoculation of the beneficial soil bacterium *Bacillus subtilis* GB03 supplemented with 0, 50, 100, or 150 mM NaCl water into soil. Growth parameters, chlorophyll content, malondialdehyde (MDA) content and osmotic potential were monitored during the growth cycle. Endogenous Na^+^ and K^+^ contents were determined at the time of harvest. White clover plants grown in GB03-inoculated soil were significantly larger than non-inoculated controls with respect to shoot height, root length, plant biomass, leaf area and chlorophyll content; leaf MDA content under saline condition and leaf osmotic potential under severe salinity condition (150 mM NaCl) were significantly decreased. Furthermore, GB03 significantly decreased shoot and root Na^+^ accumulation and thereby improved K^+^/Na^+^ ratio when GB03-inoculated plants were grown under elevated salt conditions. The results indicate that soil inoculation with GB03 promotes white clover growth under both non-saline and saline conditions by directly or indirectly regulating plant chlorophyll content, leaf osmotic potential, cell membrane integrity and ion accumulation.

## Introduction

Soil salinity has a significant negative impact on global agricultural productivity, and is a particularly acute issue in both irrigated and non-irrigated areas of the world (Flowers, 2004; Zhang et al., 2010a; Kronzucker and Britto, 2011; Zhang and Shi, 2013). Most crop and forage plants that feed the global population are sensitive to high salt concentration in soils (Rengasamy, 2010; Kronzucker et al., 2013). Soil salinity promotes osmotic stress, water deficit, stomatal closure and reduced leaf expansion (Rahnama et al., 2010; James et al., 2011); moreover, soil salinity causes deficiency of essential nutrients such as K^+^. Elevated Na^+^ inside plants can decrease plant photosynthetic rates and biomass accumulation (Mahajan and Tuteja, 2005; Munns and Tester, 2008; Zhang et al., 2010a; Zhang and Shi, 2013). Therefore, there is a need to find new ways to cope with the threat of global soil salinization to agriculture.

Beneficial interactions between bacteria and plants accelerate seed germination, promote growth, increase crop yields and secondary metabolites, and augment reproductive success. More recently plant-microbe interactions have been attributed to increased biotic and abiotic stress tolerance, including plant disease resistance and salt and drought tolerance (van Hulten et al., 2006; Zhang et al., 2008a; Hayat et al., 2010; Marques et al., 2010; Rudrappa et al., 2010; Medeiros et al., 2011; Cappellari et al., 2013; Diagne et al., 2013; de Zelicourta et al., 2013). *Bacillus subtilis* that is not toxic to humans widely exists in soils and can produce a wealth of antibacterial substances including lipopeptides, polypeptides, and phospholipids (Stein et al., 2002). Recently, Medeiros et al. (2011) found that transcriptional profiling in cotton was linked with *Bacillus subtilis* (UFLA285) which promoted biotic-stress tolerance. Zhang et al. (2011) found that a bioorganic fertilizer could effectively control banana wilt by strong colonization of *Bacillus subtilis* N11. *B. subtilis* strain GB03 that emits a complex blend of volatile organic compounds (VOCs) enhances growth and abiotic stress tolerance in *Arabidopsis* (Ryu et al., 2004; Farag et al., 2006). A bouquet of over 25 bacterial volatile odors have been identified that trigger differential expression of approximately 600 *Arabidopsis* transcripts related to cell wall modifications, primary and secondary metabolism, stress responses, hormone regulation and other expressed proteins (Zhang et al., 2007). In *Arabidopsis*, GB03 regulates auxin homeostasis and cell expansion (Zhang et al., 2007), augments photosynthesis by decreasing glucose sensing and ABA levels (Zhang et al., 2008b); promotes salt tolerance as well as reduces total Na^+^ by regulating tissue specific expression of *AtHKT1* (Zhang et al., 2008a). GB03 has also been shown to stimulate iron acquisition and increased photosynthetic capacity (Zhang et al., 2009). More recently GB03 has been found to improve osmotic-stress tolerance by elevating levels of endogenous osmoprotectants (Zhang et al., 2010b). With such beneficial plant responses activated by GB03, a legume forage crop is now being examined as to how it responds to this beneficial microbe.

White clover (*Trifolium repens* L.) is an important forage crop worldwide. Like many forage crops, white clover is sensitive to soil salinity (Rogers et al., 1997). Salt stress can decrease both above-ground growth and the number of root nodules that in turn compromise nitrogen fixation and soil fertility (Acharya et al., 2011). Since GB03 promotes growth and salt tolerance in *Arabidopsis*, the question of how GB03 improves growth and salt tolerance in the salt-sensitive forage crop white clover was addressed. The aim of current work was to evaluate the efficiency of *Bacillus subtilis* GB03 for growth promotion and salt tolerance in white clover.

## Materials and methods

### Bacterial suspension culture

*Bacillus subtilis* strain GB03 was streaked onto LB agar plates and incubated at 28°C without light for 24 h (The bacterium strain was presented by Professor Paul W. Paré at Texas Tech University, USA). Bacterial cells were then harvested from LB agar plates, transferred into liquid LB media and cultured at 28°C with 250 rpm rotation to yield 10^9^ colony forming units (CFU) mL^−1^, as determined by optical density and serial dilutions (Zhang et al., 2008a).

### Plant growth and treatments

White clover (*Trifolium repens* L. cultivar Huia) seeds were surface sterilized with 2% NaClO for 1 min followed by 70% ethanol for 10 min, and rinsed with sterile water five times (The white clover seeds was presented by Dr. Wanhai Zhou at Gansu Agricultural University, China). Seeds were then sown in presterilized plastic pots (diameter 20 cm, depth 22 cm, 10 seeds/pot with eight replicates) containing 600 g of heat-sterilized (95°C, 48 h) vermiculite and soil mix (1:1) watered with half strength Hoagland's nutrient solution (5 mM KNO_3_, 1 mM NH_4_H_2_PO_4_, 0.5 mM Ca(NO_3_)_2_, 0.5 mM MgSO_4_, 60 μM Fe-citrate, 92 μM H_3_BO_3_, 18 μM MnCl_2_·4H_2_O, 1.6 μM ZnSO_4_·7H_2_O, 0.6 μM CuSO_4_·5H_2_O, and 0.7 μM (NH_4_)_6_Mo_7_O_24_·4H_2_O). Each pot was watered with nutrient solution (400 mL) once per week. After germination, six uniform seedlings per pot were selected for continued growth and inoculated directly to the soil with 1 mL bacterial suspension culture as inoculation treatment or 1 mL liquid LB medium as a control. For salt stress treatments, seedlings were watered with 0, 50, 100, and 150 mM NaCl present in the nutrient solution. Each treatment contained eight pots as replications. Plants were grown in a glasshouse at the temperature regulated to around 28°C during the day and 22°C at night. Relative humidity averaged 65 and 75% for day and night periods, respectively, with natural photoperiod and light intensity.

### Plant biomass and physiological measurements

Sixty-day old plants were used for plant growth measurements and physiological index determination. Whole plant leaves were harvested to count leaf number per plant, measure leaf area using a leaf area meter (Epson Perfection 4870 Photo scanner, Epson America Inc., Long Beach, CA, USA) (Ma et al., 2012). Average area per leaf was calculated from leaf area per plant divided by leaf number per plant.

Plants were removed from the pots and roots were water rinsed to remove attached soil. Shoot height and root length (root depth) were measured by a ruler. Then, root and shoot were separated and blotted gently. Fresh weights were determined immediately and samples were oven dried at 80°C for 2 day for dry weight measurements.

Leaf chlorophyll content was estimated according to Porra et al. (1989). Fresh leaf samples were ground thoroughly with 80% acetone in the dark and centrifuged at 9000 g for 10 min at 4°C. Absorbance reading (UV-2102C Spectrophotometer, Unico Instrument Co., Ltd, Shanghai, China) at 645 and 663 nm for collected supernatant was used to estimate total chlorophyll content based on chlorophyll equations of Porra et al. (1989).

To probe oxidative stress the biomarker malondialdehyde (MDA) was extracted and measured spectrophotometrically using a thiobarbituric acid (TBA) protocol (Bao et al., 2009). Absorbance was determined at 450, 532, and 600 nm using a UV spectrophotometer (UV-2102C, Unico Instrument Co., Ltd, Shanghai, China).

Leaf osmotic potential (Ψs) was measured according to Ma et al. (2012). Fresh leaf samples were frozen in liquid nitrogen. Cell sap was collected by thawing slowly and then Ψs was determined using a cryoscopic osmometer (Osmomat-030, Gonotec GmbH, Berlin, Germany) at 25°C. The readings (mol·L^−1^) were used to calculate the solute potential (Ψs) in MPa with the formula Ψs = − The readings × *R* × *T*, here *R* = 0.008314 MPa · L · mol^−1^ · K^−1^ and *T* = 298.8 K (Ma et al., 2012).

### Ion analysis

Na^+^ and K^+^ contents were measured according to the method described by Wang et al. (2007). Roots were washed twice for eight min in ice-cold 20 mM CaCl_2_ to exchange cell wall-bound K^+^ and Na^+^ and the shoots were rinsed in deionized water to remove surface salts. Root and shoot were separated and samples oven dried at 70°C for 2 day. Na^+^ and K^+^ were extracted from dried plant tissues in 100 mM acetic acid at 90°C for 2 h. Ion analysis was performed using an atomic absorption spectrophotometer (2655-00, Cole-Parmer Instrument Co., Vernon Hills, IL, USA).

### Data analysis

Results of the growth, physiological index, ion contents and ion ratio were presented as means with standard deviations (*n* = 8). Statistical analyses, One-Way ANOVA and Duncan's multiple range tests, were performed.

## Results

### *Bacillus subtilis* GB03 promoted white clover growth

**Figure 1:**
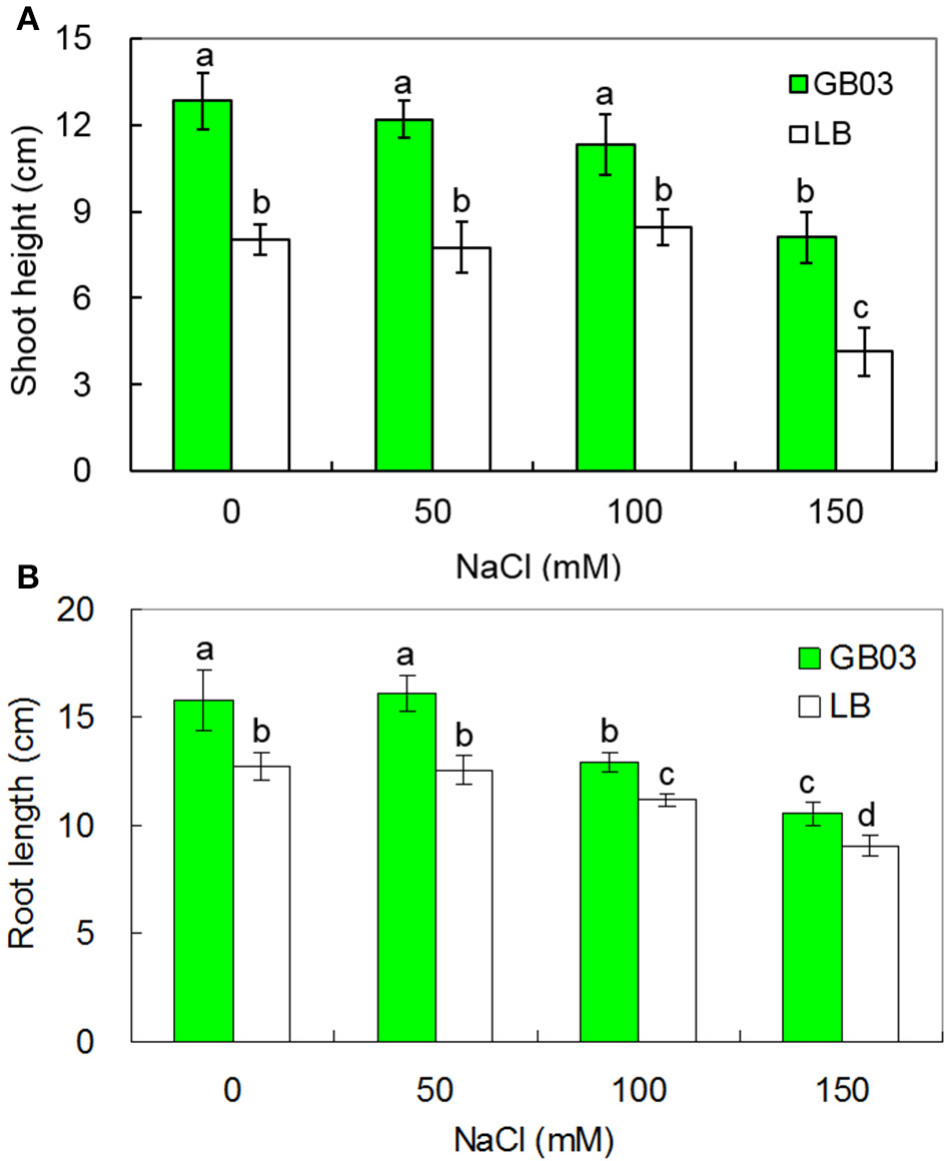
**Effects of GB03 bacterization on shoot height (A) and root length (B) of white clover under various concentrations of NaCl**. Values are means and bars indicate SDs (*n* = 8). Columns with different letters indicate significant difference at *P* < 0.05 (Duncan test).

*Bacillus subtilis* GB03 enhanced both shoot height and root length of white clover under both non-saline conditions and salinity stress (Figure 1 and Supplementary Figure 1). Shoot height was increased by 60.1% (*P* < 0.05) under non-saline conditions; compared to corresponding media control, GB03 significantly improved shoot height by 57.4% (*P* < 0.01), 33.7% (*P* < 0.05), and 95.6% (*P* < 0.01) at 50, 100, and 150 mM NaCl treatments, respectively (Figure 1A). Root length was increased by 23.9% (*P* < 0.05) under non-saline conditions; compared to corresponding media control, GB03 significantly improved root length by 28.0% (*P* < 0.01), 15.4% (*P* < 0.01), and 16.9% (*P* < 0.05) with 50, 100, and 150 mM NaCl treatments, respectively (Figure 1B).

**Figure 2:**
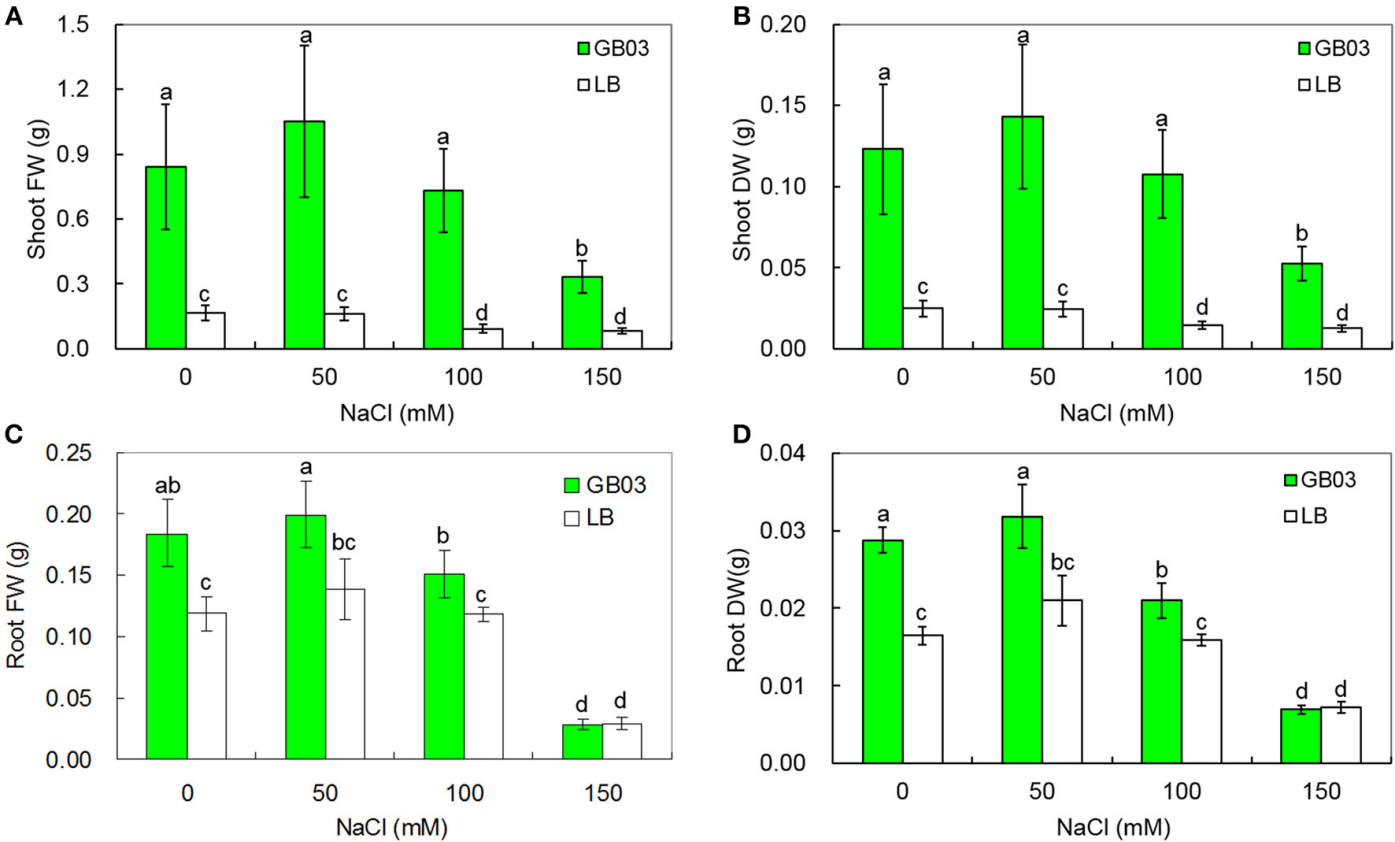
**Effects of GB03 bacterization on plant growth of white clover under various concentrations of NaCl. (A)** shoot fresh weight, **(B)** shoot dry weight, **(C)** root fresh weight, and **(D)** root dry weight. Values are means and bars indicate SDs (*n* = 8). Columns with different letters indicate significant difference at *P* < 0.05 (Duncan test).

GB03 increased plant biomass of white clover under both non-saline conditions and salinity stress (Figure 2). Shoot fresh weight was increased 4.1-fold (*P* < 0.05) under non-saline conditions; compared to corresponding media control, GB03 significantly improved shoot fresh weight by 5.5-fold (*P* < 0.05), 6.9-fold (*P* < 0.01), and 3.0-fold (*P* < 0.05) with 50, 100, and 150 mM NaCl treatment, respectively (Figure 2A). Shoot dry weight was increased by 4.0-fold (*P* < 0.05) under non-saline conditions; compared to corresponding media control, GB03 significantly improved shoot dry weight by 4.9-fold (*P* < 0.05), 6.4-fold (*P* < 0.01), and 3.2-fold (*P* < 0.05) with 50, 100, and 150 mM NaCl treatment, respectively (Figure 2B). Root fresh weight was increased by 55.0% (*P* < 0.05) under non-saline conditions; compared to corresponding media control, GB03 significantly improved shoot dry weight by 44.1% (*P* < 0.05) and 27.7% (*P* < 0.05) at 50 and 100 mM NaCl treatment, respectively (Figure 2C). Root dry weight was increased by 74.7% (*P* < 0.01) under non-saline conditions; compared to corresponding media control, GB03 significantly improved shoot dry weight by 51.7% (*P* < 0.01) and 32.5% (*P* < 0.05) at 50 and 100 mM NaCl treatment, respectively (Figure 2D). GB03 had no significant effects on root fresh and dry weights under 150 mM NaCl stress.

### GB03 increased leaf area and chlorophyll content

**Figure 3:**
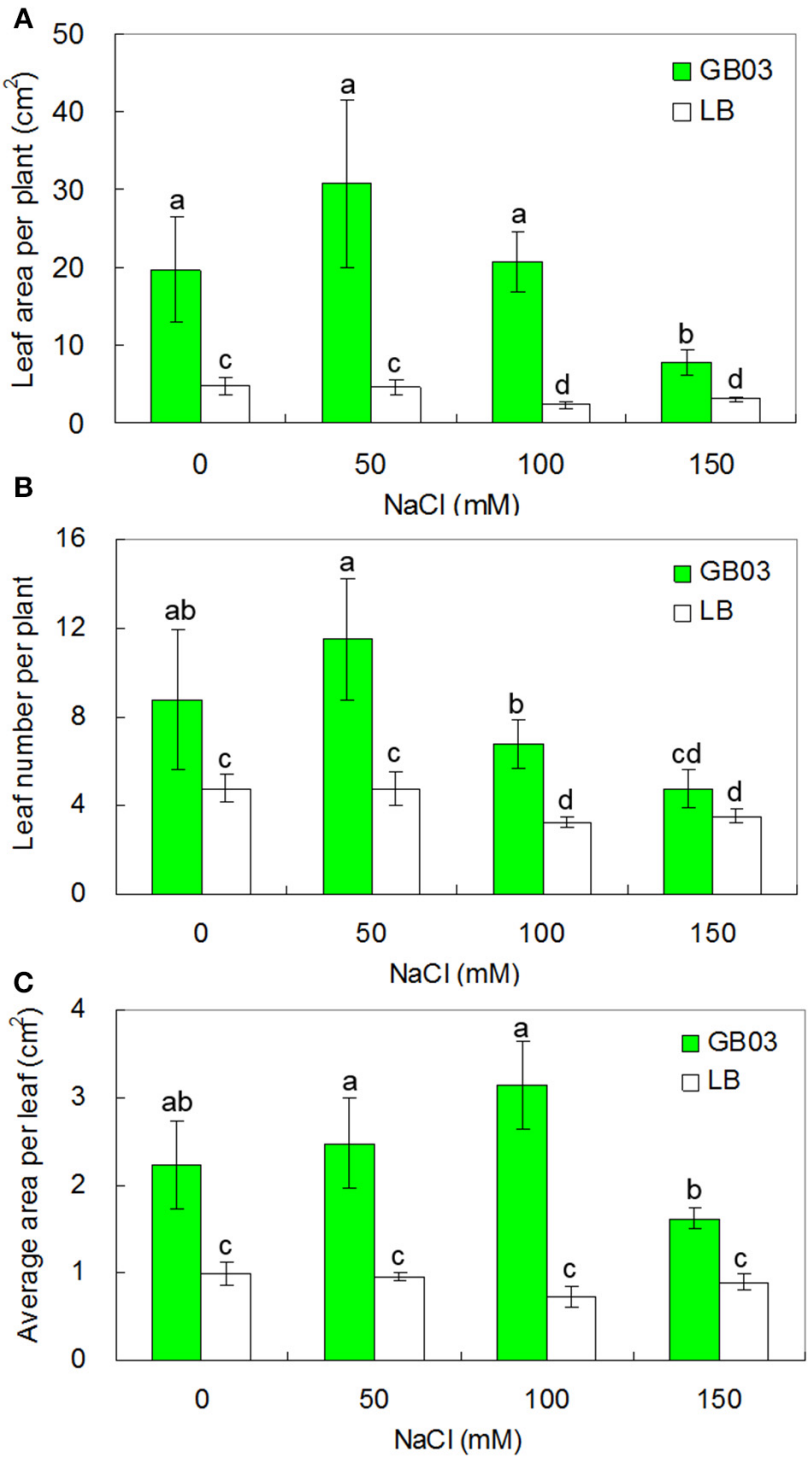
**Effects of GB03 bacterization on leaf development and growth of white clover under various concentrations of NaCl**. **(A)** leaf area per plant, **(B)** leaf number per plant, and **(C)** average area per leaf. Values are means and bars indicate SDs (*n* = 8). Columns with different letters indicate significant difference at *P* < 0.05 (Duncan test).

GB03 promoted leaf growth under both non-saline and salinity stress conditions (Figure 3). Leaf area per plant was increased by 3.1-fold (*P* < 0.05) under non-saline conditions; compared to corresponding media control, GB03 significantly increased leaf area per plant by 5.7-fold (*P* < 0.05), 7.8-fold (*P* < 0.01), and 1.5-fold (*P* < 0.05) with 50, 100, and 150 mM NaCl treatment, respectively (Figure 3A). Leaf number per plant was increased by 84.2 % (*P* < 0.05) under non-saline conditions; compared to corresponding media control, GB03 significantly elevated leaf number per plant by 142.1% (*P* < 0.05) and 107.7% (*P* < 0.05) with 50 and 100 mM NaCl treatments, respectively (Figure 3B). Average area per leaf was increased by 125.6% (*P* < 0.05) under non-saline conditions; compared to corresponding media control, GB03 significantly improved average area per leaf by 159.4% (*P* < 0.01), 334.5% (*P* < 0.01), and 81.5% (*P* < 0.01) with 50, 100, and 150 mM NaCl treatments, respectively (Figure 3C).

**Figure 4:**
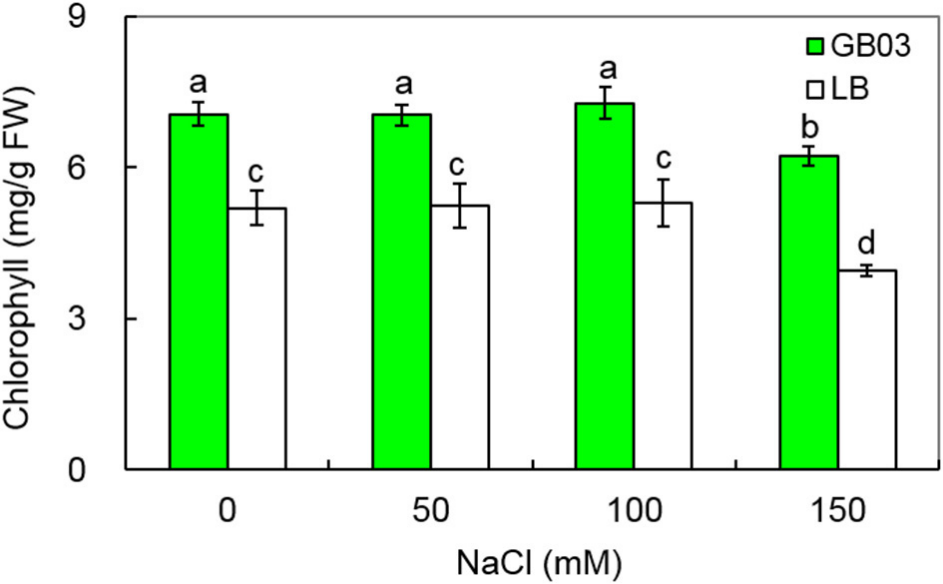
**Effects of GB03 bacterization on total chlorophyll content of white clover under various concentrations of NaCl**. Values are means and bars indicate SDs (*n* = 8). Columns with different letters indicate significant difference at *P* < 0.05 (Duncan test).

In addition to promoting leaf growth, *Bacillus subtilis* GB03 increased leaf chlorophyll content under both non-saline and salinity stress (Figure 4). Leaf chlorophyll content rose by 36.0% (*P* < 0.01) under non-saline condition; compared to corresponding media control, GB03 significantly increased leaf chlorophyll content by 34.3% (*P* < 0.01), 37.5% (*P* < 0.01), and 57.4% (*P* < 0.01) with 50, 100, and 150 mM NaCl treatments, respectively (Figure 4).

### GB03 reduced leaf osmotic potential under higher salinity condition

**Figure 5:**
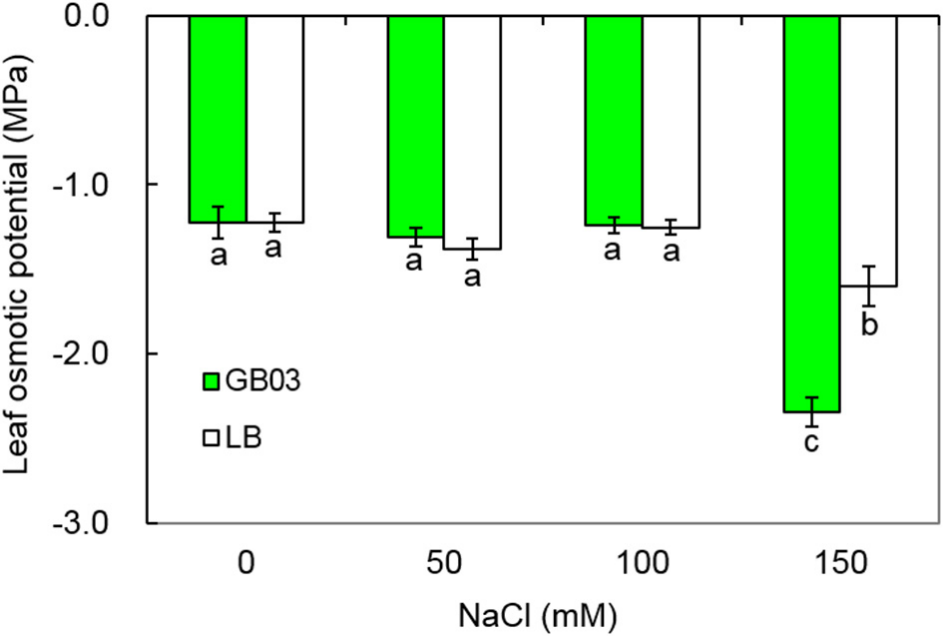
**Effects of GB03 bacterization on leaf osmotic potential of white clover under various concentrations of NaCl**. Values are means and bars indicate SDs (*n* = 8). Columns with different letters indicate significant difference at *P* < 0.05 (Duncan test).

At lower salt concentration (0, 50, and 100 mM NaCl), compared with the controls, GB03 had no significant effect on leaf osmotic potential; however, under higher salt concentration (150 mM NaCl), GB03 significantly decreased leaf osmotic potential of white clover by 46.7% (*P* < 0.05) (Figure 5).

### GB03 reduced leaf malondialdehyde (MDA) content under salinity conditions

**Figure 6:**
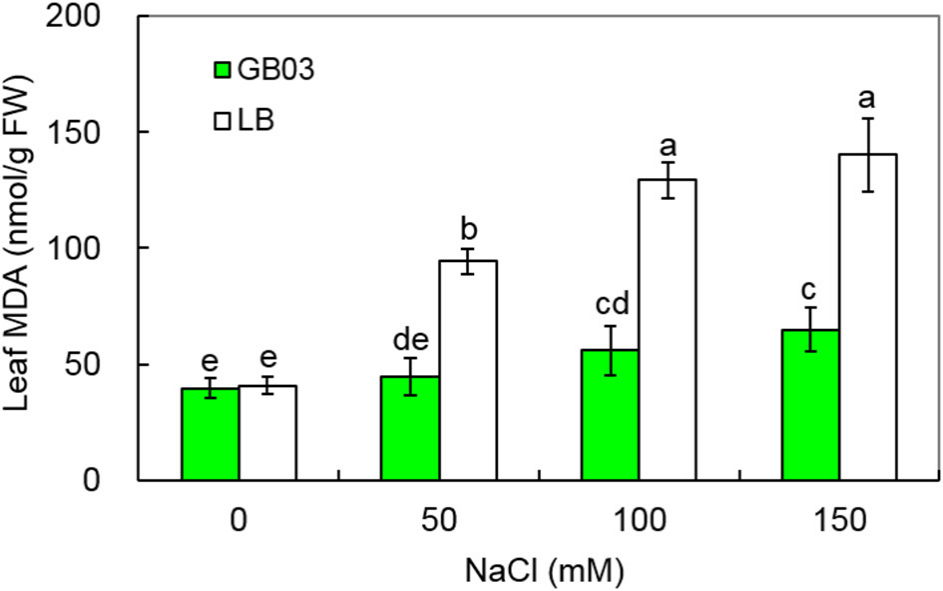
**Effects of GB03 bacterization on leaf malondialdehyde (MDA) content of white clover under various concentrations of NaCl**. Values are means and bars indicate SDs (*n* = 8). Columns with different letters indicate significant difference at *P* < 0.05 (Duncan test).

The results indicated that GB03 reduced leaf MDA content under both non-saline conditions and salinity stress (Figure 6). Leaf MDA content was not changed under non-saline conditions; however, compared to corresponding media control, GB03 significantly reduced leaf MDA content by 52.7% (*P* < 0.01), 56.8% (*P* < 0.01), and 53.8% (*P* < 0.05) with 50, 100, and 150 mM NaCl treatments, respectively (Figure 6), thus, oxidative stress tolerance and integrity of the cell membrane as measured by the biomarker MDA were both favorably regulated by GB03.

### GB03 reduced sodium accumulation under salinity conditions

**Figure 7:**
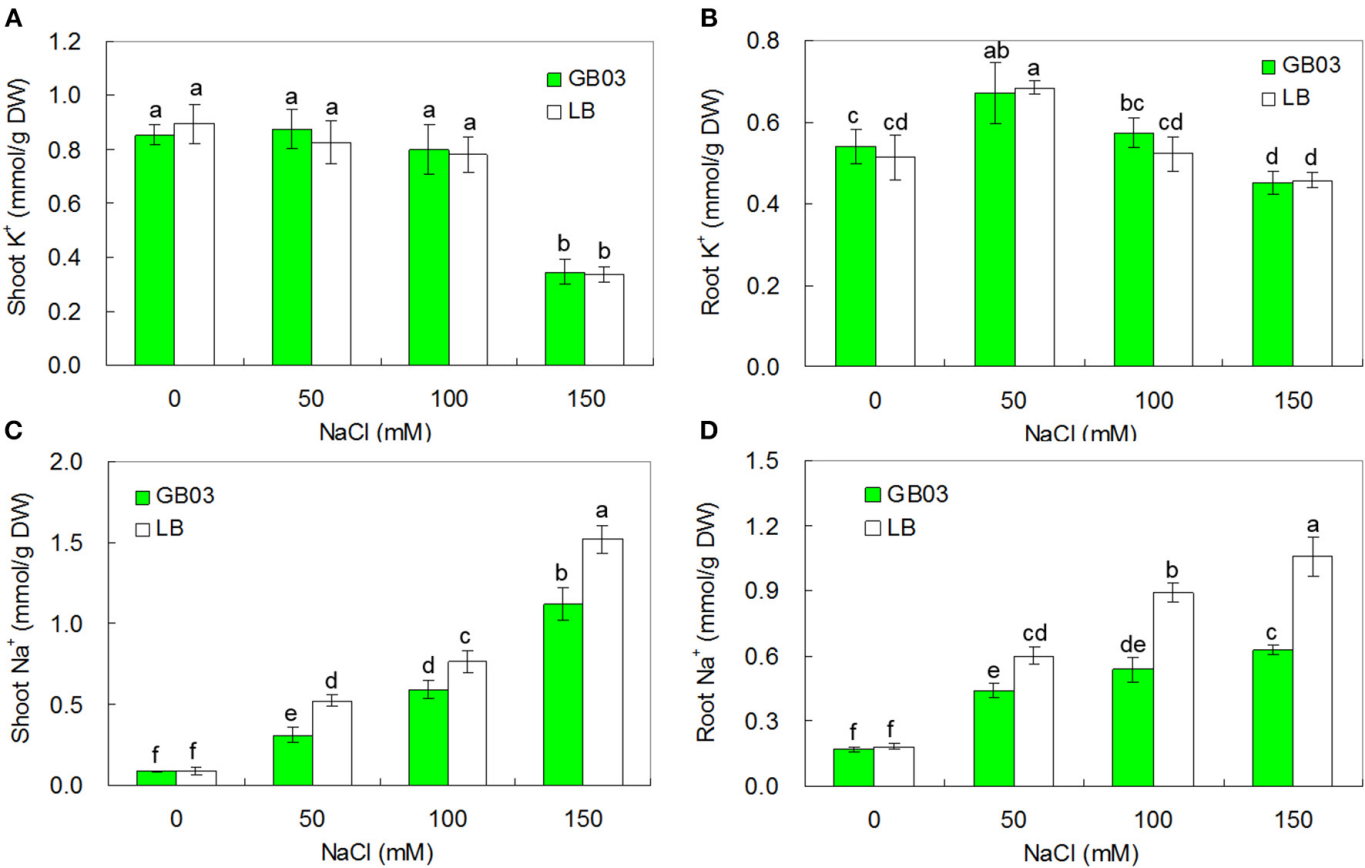
**Effects of GB03 bacterization on ion contents of white clover under various concentrations of NaCl. (A)** shoot K^+^ content, **(B)** root K^+^ content, **(C)** shoot Na^+^ content, **(D)** root Na^+^ content. Values are means and bars indicate SDs (*n* = 8). Columns with different letters indicate significant difference at *P* < 0.05 (Duncan test).

To test whether GB03 inoculation resulted in altered ion accumulation in white clover under various salinity conditions, endogenous Na^+^ and K^+^ content was measured in the 0, 50, 100, and 150 mM NaCl treatments. K^+^ accumulation was un-effected by GB03 inoculation (Figures 7A,B). However, endogenous Na^+^ accumulation was reduced significantly. Compared to the media control, GB03 significantly decreased shoot Na^+^ content by 40.7% (*P* < 0.01), 22.5% (*P* < 0.01), and 26.3% (*P* < 0.01), as well as root Na^+^ content by 27.1% (*P* < 0.05), 39.7% (*P* < 0.01), and 40.7% (*P* < 0.01) with 50, 100, and 150 mM NaCl treatments, respectively (Figures 7C,D).

K^+^/Na^+^ ratios were also significantly improved by GB03 inoculation with 50, 100, and 150 mM NaCl treatments. Compared to corresponding media control, Shoot K^+^/Na^+^ ratios increased by 78.6% (*P* < 0.01), 32.0% (*P* < 0.05), and 39.7% (*P* < 0.05) as well as root K^+^/Na^+^ ratios by 34.6% (*P* < 0.05), 82.2% (*P* < 0.01), and 66.2% (*P* < 0.01) under soil salinity conditions of 50, 100, and 150 mM NaCl, respectively (Table 1).

**Table 1:** **Effects of GB03 bacterization on K^+^/Na^+^ ratio in shoot and root of white clover under various concentrations of NaCl**.

**NaCl (mM)**	**Shoots**		**Roots**	
	**GB03**	**LB**	**GB03**	**LB**
0	9.87 ± 0.42a	9.81 ± 0.80a	3.13 ± 0.25a	2.81 ± 0.30a
50	2.81 ± 0.24b	1.58 ± 0.15c	1.53 ± 0.17b	1.14 ± 0.03c
100	1.35 ± 0.15cd	1.03 ± 0.09d	1.07 ± 0.07c	0.59 ± 0.05e
150	0.31 ± 0.04e	0.22 ± 0.02e	0.72 ± 0.05d	0.43 ± 0.02f

*Values are means ± SDs (n = 8). Different letters indicate significant difference at P < 0.05 (Duncan test) within shoots and roots, individually*.

## Discussion

### Influence of *Bacillus subtilis* GB03 on growth of white clover under saline condition

Beneficial soil bacteria promoted plant growth of many plant species (Bashan et al., 2000; Gray and Smith, 2005; Xie et al., 2009; Paré et al., 2011). Under salinity stress, inducible plant growth promotion mediated by beneficial soil bacteria has also been observed in several cultivated and wild plant species including dwarf saltwort (*Salicornia bigelovii*) (Bashan et al., 2000), tomato (*Lycopersicon esculentum*) (Mayak et al., 2004), chickpea (*Cicer arietinum*) (Mhadhbi et al., 2004), alfalfa (*Medicago sativa*) (Ibragimova et al., 2006), common glasswort (*Salicornia europea*) (Ozawa et al., 2007), maize (*Zea mays*) (Bano and Fatima, 2009), and wheat (*Triticum aestivum*) (Tiwari et al., 2011). The growth promotion of white clover by GB03 inoculation in soil evaluated in the present study is consistent with previous reports in *Arabidopsis* (Zhang et al., 2007, 2008a,b, 2010b; Xie et al., 2009; Paré et al., 2011). Interestingly, our results indicated that GB03 is a more efficient promoter of shoot growth than root growth in white clover, especially under salt exposure (50, 100, and 150 mM NaCl). Whether GB03 promotes growth of white clover by enhancing nitrogen fixation and the coordination between nitrogen-fixing bacteria and *Bacillus subtilis* (GB03) in the rhizosphere remain to be investigated.

Leaf development plays an important role in plant production since it affects the area available for photosynthesis, which is strongly related to plant growth and biomass accumulation (Gutierrez-Boem and Thomas, 1998; Battie-Laclau et al., 2013). In our study, GB03 inoculation significantly increased leaf area per plant, leaf number per plant, and thereby average area per leaf in white clover. Leaf chlorophyll content is also an important physiological trait directly linked to photosynthesis rate in plants (Ma et al., 2012). Previous studies showed that plants grown under salinity conditions produced less chlorophyll and dry matter than those without salinity stress due to chlorophyll peroxidation (Hernandez et al., 1995; Zayed and Zeid, 1998; Lunde et al., 2007; Tuna et al., 2008; Barry, 2009). Zhang et al. (2008b) found that GB03 augments photosynthetic capacity by increasing photosynthetic efficiency and chlorophyll content in *Arabidopsis*. With white clover, GB03 significantly improved leaf chlorophyll content under both non-saline and salinity conditions (50, 100, and 150 mM NaCl).

### GB03 ameliorated leaf osmotic adjustment ability and alleviated cell membrane damage under saline conditions

When plants are exposed to saline or drought conditions, osmotic stress rapidly follows (Munns and Tester, 2008). Salt stress reduces the rates of photosynthesis and transpiration, stomatal conductance and leaf area etc. (Wang et al., 2005; Ramani et al., 2006). Leaves could accumulate abundant osmoprotectant and adjust their osmotic potential (≈leaf water potential) below that of the apoplast and soil in order to ensure that the plants can continue to absorb moisture from the soil and maintain turgor pressure, thus improving their stress tolerance (Bao et al., 2009; Jha et al., 2011; Janz and Polle, 2012; Ma et al., 2012).

Zhang et al. (2010b) reported that GB03 enhanced *Arabidopsis* choline and glycine betaine synthesis associated with enhanced osmolyte content, resulting in increased plant tolerance to osmotic stress. In the case of white clover, GB03 significantly reduced leaf osmotic potential under severe salt stress conditions (150 mM NaCl).

Soil salinity is known to increase the level of reactive oxygen species in plant leaves, which are well recognized for membrane lipid peroxidation and cause an increase in leaf malondialdehyde (MDA), a product of membrane lipid peroxidation (Koca et al., 2006; Yazici et al., 2007). Therefore, leaf MDA content, representing the degree of cell membrane damage, is usually used to evaluate plant tolerance to salinity and drought (Luna et al., 2000; Miao et al., 2010). In this work, GB03 significantly decreased leaf MDA content of white clover under saline conditions (50, 100, and 150 mM NaCl).

### GB03 reduced Na^+^ content in plants

Saline soils contain a varied and complex array of cation-anion pairs (e.g., Na_2_SO_4_, MgSO_4_, CaSO_4_, MgCl_2_, KCl, and Na_2_CO_3_), with Na^+^ often the dominant species (Zhang et al., 2010a). Growth inhibition, a common plant response to soil salinity, is correlated with high internal Na^+^ concentration and low K^+^/Na^+^ ratio in the plant (Zhang et al., 2010a). Plants grown in saline conditions can minimize Na^+^ toxicity by restricting Na^+^ uptake and Na^+^ xylem loading, extruding Na^+^ from root cells and redirecting Na^+^ from shoots to roots (Tester and Davenport, 2003; Munns and Tester, 2008; Zhang et al., 2010a; Kronzucker and Britto, 2011; Zhang and Shi, 2013). Zhang et al. (2008a) found that in *Arabidopsis* GB03 decreased whole plant Na^+^ content to 54% of that in control plants by down-regulating *HKT1* expression in roots to decrease root Na^+^ uptake and up-regulating *HKT1* expression in shoots to enhance shoot-to-root Na^+^ recirculation, respectively. In this study, GB03 soil inoculation significantly decreased Na^+^ accumulation and increased K^+^/Na^+^ in both shoots and roots of white clover under salt stress with no measurable effect on K^+^ content in the whole plant. Whether GB03 improves salt tolerance of white clover by tissue specific regulation of *HKT* gene expression remains to be investigated.

In summary, the results presented here established that the inoculation of the soil bacterium *Bacillus subtilis* GB03 to the rhizosphere significantly increases plant growth and biomass of the forage legume white clover under both non-saline and saline conditions. GB03-regulated plant processes include chlorophyll abundance, leaf osmotic potential, cell membrane integrity, and ion accumulation. This study provides insight for the application of selected bacteria to cultivated legumes to combat saline toxicity.

### Conflict of interest statement

The authors declare that the research was conducted in the absence of any commercial or financial relationships that could be construed as a potential conflict of interest.

## Supplementary material

Supplementary Figure 1 **Effects of bacterization on growth of white clover plants in pots under various concentrations of NaCl.** Here GB03 represents *Bacillus subtilis* GB03 suspension culture in LB medium, LB Luria Broth medium without bacteria and 0, 50, 100, and 150 the concentrations of NaCl (mM).
