# Supplementary figures and images for: Beneficial soil bacterium Bacillus subtilis (GB03) augments salt tolerance of white clover
Source: Front Plant Sci. 2014 Oct 8;5:525. doi: 10.3389/fpls.2014.00525 (PMC4189326; doi:10.3389/fpls.2014.00525)

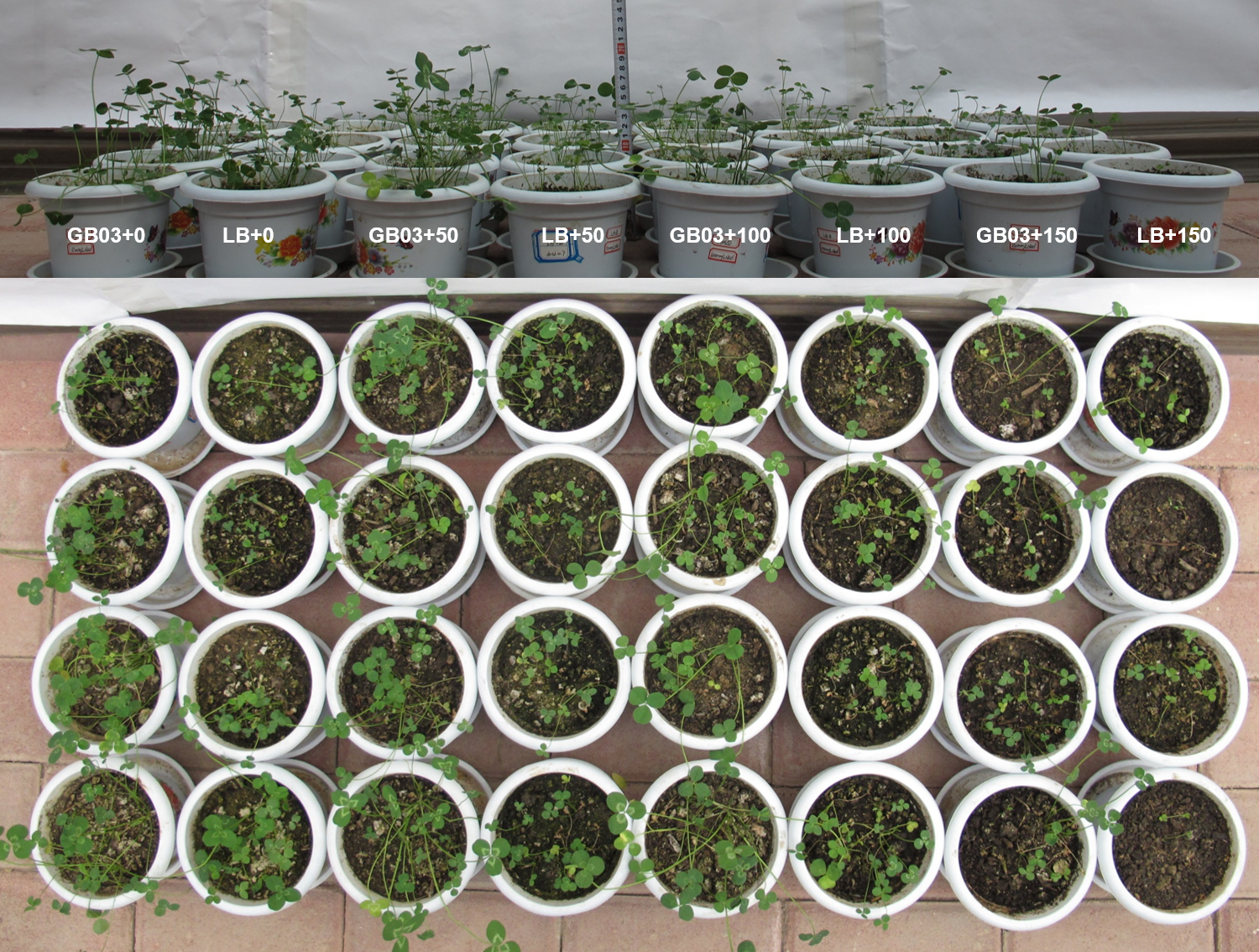

Supplement: Supplementary Figure 1 — Effects of bacterization on growth of white clover plants in pots under various concentrations of NaCl. Here GB03 represents Bacillus subtilis GB03 suspension culture in LB medium, LB Luria Broth medium without bacteria and 0, 50, 100, and 150 the concentrations of NaCl (mM). [file Image1.TIF]
